# Quality Control of Motor Unit Number Index (MUNIX) Measurements in 6 Muscles in a Single-Subject “Round-Robin” Setup

**DOI:** 10.1371/journal.pone.0153948

**Published:** 2016-05-02

**Authors:** Christoph Neuwirth, Christian Burkhardt, James Alix, José Castro, Mamede de Carvalho, Malgorzata Gawel, Stephan Goedee, Julian Grosskreutz, Timothée Lenglet, Cristina Moglia, Taha Omer, Maarten Schrooten, Markus Weber

**Affiliations:** 1 Neuromuscular Diseases Unit / ALS Clinic, Kantonsspital St.Gallen, St.Gallen, Switzerland; 2 Sheffield Institute for Translational Neuroscience, University of Sheffield, Sheffield, England; 3 Department of Neurosciences, Hospital de Santa Maria, Instituto de Medicina Molecular, Faculty of Medicine, University of Lisbon, Lisbon, Portugal; 4 Department of Neurology, Medical University of Warsaw, Warsaw, Poland; 5 Brain Centre Rudolf Magnus, Department of Neurology and Neurosurgery, UMC Utrecht, Utrecht, The Netherlands; 6 Hans-Berger Department of Neurology, University Hospital Jena, Jena, Germany; 7 Département de Neurophysiologie, Groupe hospitalier Pitié-Salpêtrière, APHP, Paris, France; 8 ALS Center of Torino, Department of Neuroscience "Rita Levi Montalcini", University of Torino, Torino, Italy; 9 Trinity College Biomedical Science Institute (TBSI) and Beaumont Hospital, Dublin, Ireland; 10 Department of Neurology, University Hospital Leuven, Leuven, Belgium; 11 Department of Neurology, University Hospital Basel, Basel, Switzerland; University of Sydney, AUSTRALIA

## Abstract

**Background:**

Motor Unit Number Index (MUNIX) is a neurophysiological measure that provides an index of the number of lower motor neurons in a muscle. Its performance across centres in healthy subjects and patients with Amyotrophic Lateral Sclerosis (ALS) has been established, but inter-rater variability between multiple raters in one single subject has not been investigated.

**Objective:**

To assess reliability in a set of 6 muscles in a single subject among 12 examiners (6 experienced with MUNIX, 6 less experienced) and to determine variables associated with variability of measurements.

**Methods:**

Twelve raters applied MUNIX in six different muscles (abductor pollicis brevis (APB), abductor digiti minimi (ADM), biceps brachii (BB), tibialis anterior (TA), extensor dig. brevis (EDB), abductor hallucis (AH)) twice in one single volunteer on consecutive days. All raters visited at least one training course prior to measurements. Intra- and inter-rater variability as determined by the coefficient of variation (COV) between different raters and their levels of experience with MUNIX were compared.

**Results:**

Mean intra-rater COV of MUNIX was 14.0% (±6.4) ranging from 5.8 (APB) to 30.3% (EDB). Mean inter-rater COV was 18.1 (±5.4) ranging from 8.0 (BB) to 31.7 (AH). No significant differences of variability between experienced and less experienced raters were detected.

**Conclusion:**

We provide evidence that quality control for neurophysiological methods can be performed with similar standards as in laboratory medicine. Intra- and inter-rater variability of MUNIX is muscle-dependent and mainly below 20%. Experienced neurophysiologists can easily adopt MUNIX and adequate teaching ensures reliable utilization of this method.

## Introduction

Motor Unit Number Index (MUNIX) is a novel variant of motor unit number estimation (MUNE) techniques which provides an index of the number of functional lower motor neurons in a muscle. Recent studies have suggested that this technique may serve as a marker of disease progression in diseases with progressive loss of motor units, such as amyotrophic lateral sclerosis (ALS). Several studies have also demonstrated a good test-retest reliability in healthy subjects and ALS patients. [1–7]

Sensitive biomarkers in early phase II ALS trials are sorely needed to reveal potential beneficial effects of therapeutic interventions. [8] Biomarkers directly linked to the fundamental underlying disease process, which in the case of ALS is the loss of motor neurons over time, would be advantageous. An important attribute of any biomarker is not only its sensitivity to change, but also reliability of measurements, which will allow a reduction in sample size and increase power to detect significant differences in ALS trials. [9] In laboratory medicine assessment of inter-centre variability can be relatively easily achieved with so called “round robin” tests. [10] A well-defined sample is sent to different laboratories which then perform a test-retest and compare the results with a reference value.

This kind of quality control is difficult to achieve in outcome measures or biomarkers which are linked to the performance of the test subject and/or rater. However, a pivotal study of the forced vital capacity in a large multi-centre trial clearly showed that after adequate training an excellent inter-rater reliability can be achieved. [11] Such an approach has never been used for neurophysiological measures or neuroimaging. Over the past few years, several European centres have been trained to undertake the novel MUNIX method as part of the SOPHIA (Sampling and biomarker OPtimization and Harmonization In ALS and other motor neuron diseases) project. A refresher course held during the ENCALS meeting in Dublin 2015 offered a unique opportunity to perform a “round robin” test on a single subject. The goal was to evaluate MUNIX variability among 12 raters and to analyse associated factors.

## Subjects and Methods

At the ENCALS (European Network for the Cure of ALS) meeting in Dublin 2015, a MUNIX training course was held over 2 days. Neurophysiologists from different European countries already familiar with this method and who had previously attended one or more training courses were invited. Twelve raters were included, 6 of them had passed a qualification process as part of a longitudinal study (SOPHIA). For this qualification process, raters had to perform MUNIX measurements in 6 muscles (*Mm*. *abductor digiti minimi*, *abductor pollicis brevis*, *biceps brachii*, *tibialis anterior*, *extensor digitorum brevis* and *abductor hallucis*) in 4 healthy volunteers in two separate sessions. Raw data and results were sent to one reviewer (C.N.) and raters were certified when measurements showed a coefficient of variation (COV) below 20%.

During the round robin study, all 12 raters measured above mentioned muscles in one healthy subject (M.W.) in two sessions. Test and retest session were separated by one day. No specific sequence of raters was determined but the order was kept the same on the 2 consecutive days. A Dantec Keypoint^®^ Focus EMG system was used with clamp cables and self-adhesive Kendall ™ Nutab electrodes with 15 mm diameter for recordings. Electrodes and marks were completely removed between each rater. Raters were timed during the recording and allowed a maximum of 5 minutes on a single muscle. MUNIX values of recordings were calculated separately after the recording process.

MUNIX applies a statistical approach, using the area and power of the supramaximal stimulated compound muscle action potential (CMAP) and area and power of the surface electromyography with different force levels of voluntary isometric activation. With these values the ‘ideal case motor unit count’ is computed to estimate the amount motor neurons, reflected by an index value. The method has been described in detail. [3, 12]

Electrode placement and electrical supramaximal nerve stimulation was performed according to standard neurographic procedures. A mandatory step was to reposition the recording electrode over the muscle belly several times to obtain the highest CMAP amplitude. Details of electrode placements including photo material are available online at http://www.encals.eu/page/european-collaborative-projects.

The protocol for MUNIX test-retest measurements in healthy volunteers was approved by the Ethics Committee St.Gallen previously. [6] The single test subject (M.W.) gave written informed consent to participate during the ENCALS meeting and MUNIX training course. According to the Swiss regulations, no separate ethical approval was needed for observational single case studies in a healthy subject.

In advance of the meeting, raters were sent a questionnaire regarding their general experience in electrophysiology, percent of daily time devoted to electrophysiology and nerve conduction studies (NCS), number of prior performed MUNIX measurements, number of MUNIX training sessions undertaken and what they felt would be the two most difficult muscles to measure. Variables assessed during the MUNIX measurements included procedure time and maximum electric stimulation intensity for each single measurement.

Since a systematic error (e.g. non-optimal CMAP amplitude) may not necessarily affect the test-retest reliability but accuracy, in addition a hypothetical reference value was determined for each muscle. For this reference value, the 6 largest CMAPs (mean of test-retest measurements) were determined for each muscle. Of these 6 test-retest measurements, the 3 test-retest measurements with the lowest CMAP variability were selected to calculate the “reference” CMAP amplitudes and the corresponding MUNIX values (mean of 3 measurements).

The muscle-specific difference between real measurements and reference value was determined for all raters (accuracy).

### Statistics

To evaluate the reliability of MUNIX and CMAP measurements, the coefficient of variation (COV: 100*SD/mean) and variability (VAR: 100*difference of test-retest/mean) were determined for each muscle. Intra-class correlation coefficient values turned out to be unfavourable because of the special situation of only one study subject yielding inter-subject variabilities near zero. Depending on the comparisons Welch's t-test, paired t-test, (nested) linear mixed-effects models with "Rater" as random effect and linear regressions were performed as indicated in the results.

All analyses were performed using the statistical programme R Version.2.15.2. [12]

## Results

MUNIX was well tolerated in the single subject, even when a total of 144 measurements were performed over 2 days. One rater (rater 5) was unsuccessful in obtaining a proper biceps CMAP on the first measurement. As per protocol the recording was aborted after 5 minutes. Otherwise, no major technical issues occurred.

Table 1 shows the characteristics of raters. Raw data are listed in the S1 Table.

**Table 1:** Characteristics of raters familiar (1 to 6) and less familiar (7 to12) with the MUNIX method. # = number.

	experience	daily time	NCS per	experience	MUNIX			MUNIX
	neurophys.	neurophy.	week	of MUNIX	/ muscle	challenging	challenging	courses
rater	[years]	[%]	[#]	[months]	[#]	muscle 1	muscle 2	[#]
1	12	25–50%	26–50	84	>100	AH	Biceps	4
2	9	25–50%	26–50	30	51–100	AH	TA	3
3	11	>75%	51–100	72	>100	Biceps	AH	2
4	3	>75%	11–25	17	26–50	Biceps	AH	1
5	15	50–75%	26–50	17	>100	Biceps		2
6	3	25–50%	26–50	24	>100	EDB		1
7	25	50–75%	26–50	36	11–25	AH		3
8	15	10–25%	11–25	12	1–10	EDB	Biceps	2
9	8	<10%	11–25	5	11–25	AH	Biceps	2
10	10	50–75%	26–50	36	11–25	Biceps		1
11	4	25–50%	11–25	10	26–50	EDB	Biceps	1
12	10	25–50%	51–100	24	11–25	AH	Biceps	1
mean	10.4	25–50% *	26–50 *	30.6	26–50 *			1.9
SD	(± 6.2)			(± 24.3)				(± 1.0)

* = *median*

All raters specified prior to the study which two muscles they felt to be most challenging. The biceps muscle was mentioned most frequently, followed by the M. abductor hallucis. (Table 1)

### Reliability

**Fig 1:**
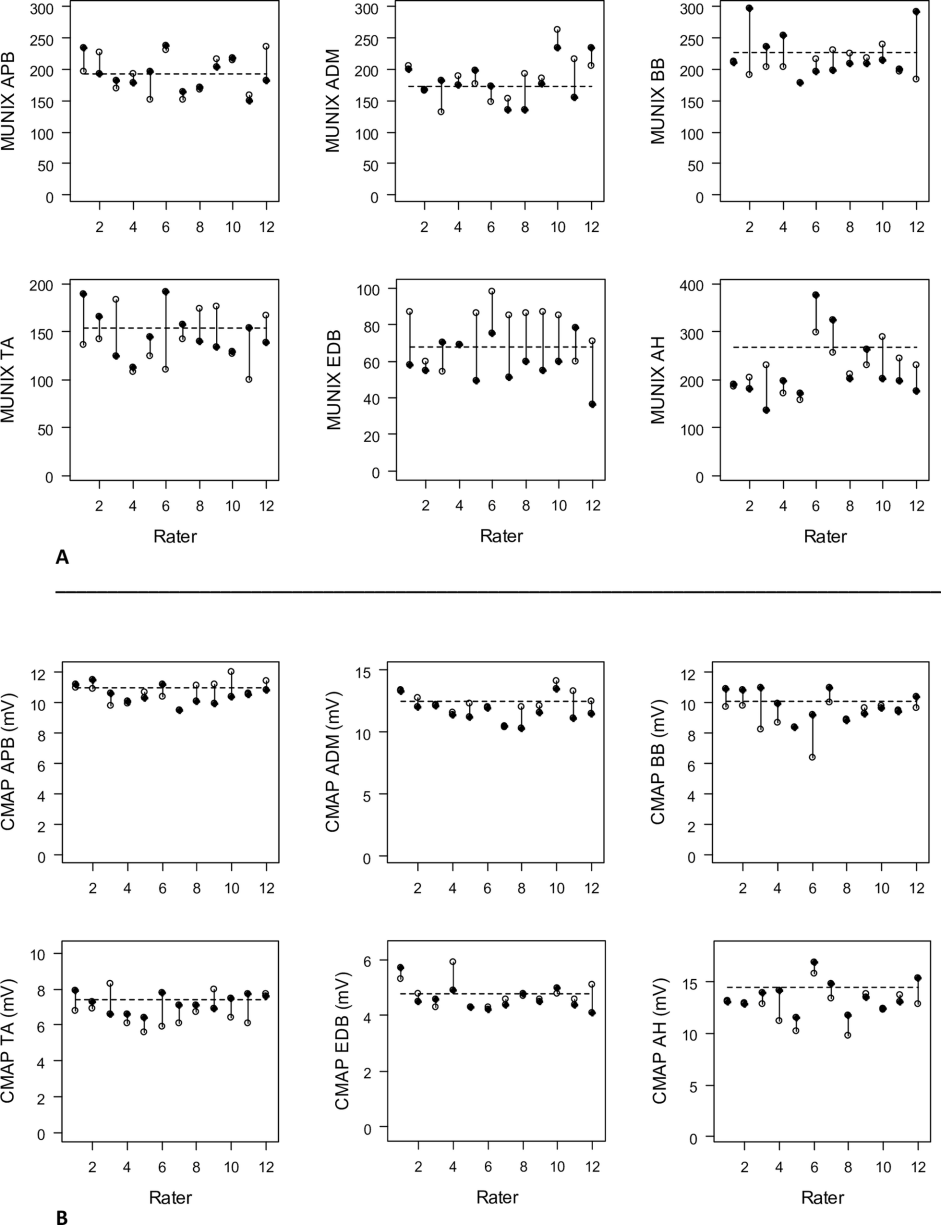
**A and B:** Test-retest results for MUNIX (A) and CMAP (mV) (B) in individual muscles. The dotted lines indicate the hypothetical reference value. Note the different y-axis scales for each muscle. Open dots = test values, filled dots = retest values.

Test-retest data and coefficients of variation (COV) for MUNIX measurements in individual muscles are summarized in Fig 1 and Tables 2 and 3, respectively.

**Table 2:** Coefficient of variation (COV) and variability () for MUNIX measurements in individual muscles in raters.

intra-rater	all raters (n = 12)		exp. raters (n = 6)		non-exp. raters (n = 6)	
muscle	COV MUNIX	COV CMAP	COV MUNIX	COV CMAP	COV MUNIX	COV CMAP
APB	7.4 (10.5)	4.2 (5.9)	9.0 (12.8)	3.3 (4.7)	5.8 (8.2)	5.0 (7.1)
ADM	10.7 (15.1)	4.2 (5.9)	8.5 (12.0)	2.4 (3.3)	12.9 (18.3)	6.0 (8.5)
BB	11.1 (15.6)	8.0 (11.3)	12.6 (17.8)	14.1 (19.9)	8.9 (13.9)	2.9 (4.2)
TA	16.6 (23.5)	9.9 (14.0)	18.9 (26.7)	10.9 (15.4)	14.4 (20.3)	9.0 (12.7)
EDB	24.3 (34.4)	4.7 (6.7)	18.4 (26.0)	4.9 (6.9)	30.3 (42.8)	4.6 (6.5)
AH	13.9 (19.6)	6.2 (8.8)	13.0 (18.4)	6.1 (8.6)	14.8 (20.9)	6.3 (8.9)

**Table 3:** Inter-rater variability (COV) in individual muscles for the first and second measurement and mean of both values.

inter-rater	1st measurement		2nd measurement		mean of measurements	
muscle	COV MUNIX	COV CMAP	COV MUNIX	COV CMAP	COV MUNIX	COV CMAP
APB	16.4	6.7	14.1	5.6	13.5	4.9
ADM	18.9	7.7	17.8	8.2	15.9	7.4
BB	8.0	11.6	16.8	9.2	8.1	8.5
TA	20.3	12.9	16.4	7.0	10.9	6.9
EDB	18.0	9.8	20.0	9.5	12.2	8.4
AH	19.2	13.0	31.7	11.4	22.8	11.3

Intra-rater coefficient of Variation (COV) ranged from 7.4 (APB) to 24.3 (EDB). Range was smaller in the experienced group (8.5 (ADM) and 18.9 (TA)) with a mean of 13.4 (SD ± 4.5) compared to the less experienced group (5.8 (APB) to 30.3 (EDB), mean 14.7 (SD ± 8.4)). The EDB showed comparably low MUNIX values (mean MUNIX 69 ± 16) compared to the MUNIX values of other 5 muscles (mean 192 ± 35), which contributes to a relatively higher COV.

Inter-rater reliability differed muscle-specific considering both measurements and ranged from 8.0 to 31.7 (mean 18.1± 5.4) (Table 3). The biceps exhibited the lowest overall inter-rater variability, the AH the largest (means of measurements).With the exception of the AH, all other muscles revealed inter-rater COV equal or below 20%.

**Fig 2:**
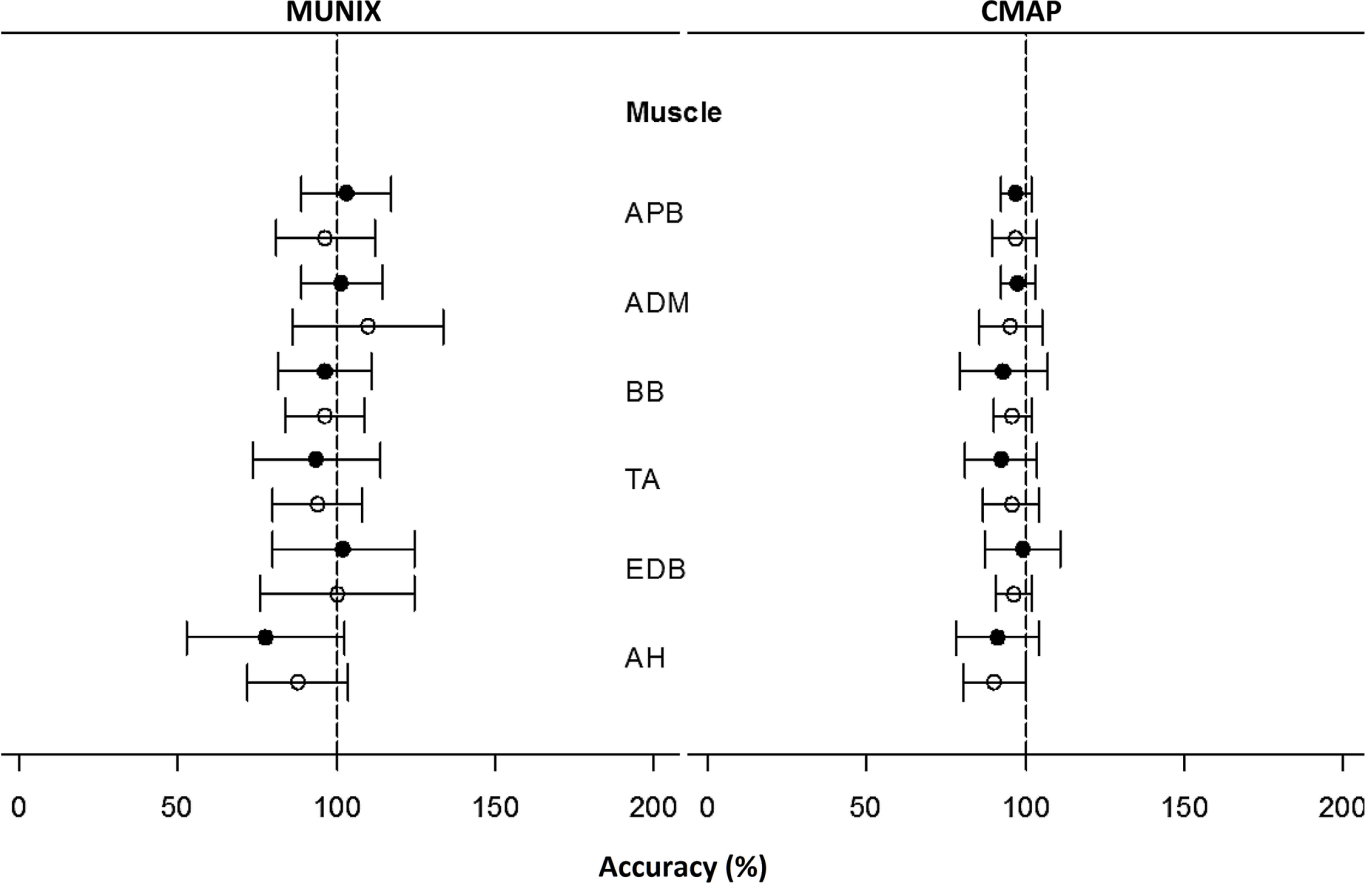
Relative mean and standard deviation of MUNIX and CMAP measurements in individual muscles of the experienced group (filled circles) and less-experienced group (empty circles) compared to the hypothetical reference values, expressed as accuracy (%).

Analysing the difference between MUNIX and CMAP measurements and the arbitrary reference value revealed a high accuracy (relative mean) and good reliability (SD) of measurements. (Fig 2)

Among the 12 individual raters, intra-rater COV of all MUNIX measurements ranged from 6.6 to 22.9 (mean 14.1 ± 4.3, data not shown).

In univariate linear mixed-effects models, no significant influence on the variability of MUNIX and CMAP measurements was present for general neurophysiological experience (years), amount of clinical electrophysiology in daily practice, experience in MUNIX (months) and number of attended MUNIX training courses (data not shown).

Between the experienced and less experienced group, no significant differences were observable for MUNIX, CMAP, time and maximum stimulation intensity determined by Welch’s t-tests (p values >0.22, not shown in Table 4). One rater in the experienced group used habitually higher stimulation intensities (up to 85 mA) compared to all other raters, leading to a trend of slightly higher stimulation intensities in the experienced group.

**Table 4:** Descriptive results of MUNIX and CMAP measurements in different rater groups.

	all raters (n = 12)				experienced raters (n = 6)				non-experienced raters (n = 6)			
muscle	MUNIX	CMAP mV	time (min)	max. stim. (mA)	MUNIX	CMAP mV	time (min)	max. stim. (mA)	MUNIX	CMAP mV	time (min)	max. stim. (mA)
APB	192 (29)	10.6 (0.7)	3.2 (1.2)	28.3 (17.7)	199 (27)	10.6 (0.5)	3.2 (1.4)	28.9 (18.1)	186 (30)	10.6 (0.8)	3.3 (1.1)	27.6 (18.0)
ADM	183 (33)	12.0 (1.0)	3.6 (1.3)	21.9 (10.7)	176 (22)	12.2 (0.7)	3.5 (1.1)	25.0 (13.0)	190 (41)	11.9 (1.3)	3.8 (1.5)	18.9 (7.1)
BB	218 (30)	9.7 (0.6)	3.5 (0.9)	34.3 (12.2)	218 (33)	9.4 (1.4)	3.8 (1.0)	39.2 (15.1)	218 (28)	9.7 (0.6)	3.3 (0.9)	29.8 (6.5)
TA	145 (26)	7.0 (0.7)	4.3 (1.4)	23.4 (9.2)	144 (31)	6.8 (0.8)	4.0 (1.1)	24.9 (11.2)	145 (22)	7.1 (0.6)	4.7 (1.7)	21.9 (6.8)
EDB	69 (16)	4.7 (0.5)	4.9 (1.6)	43.6 (14.8)	69 (15)	4.8 (0.6)	5.0 (1.9)	46.8 (19.1)	68 (17)	4.6 (0.3)	4.9 (1.4)	40.4 (8.6)
AH	222 (57)	13.1 (1.6)	4.2 (1.4)	34.9 (14.2)	208 (67)	13.2 (1.9)	4.4 (0.9	37.4 (17.0)	235 (43)	13.1 (1.5)	4.0 (1.8)	32.4 (11.0)

() = SD

Retests the following day were generally performed 0.6 minutes faster (all 6 muscles together) (p = 0.001, paired t-test). A more detailed analysis by a nested linear mixed effects model revealed that only the AH and TA differed significantly (p = 0.027 and 0.006, respectively).

**Fig 3:**
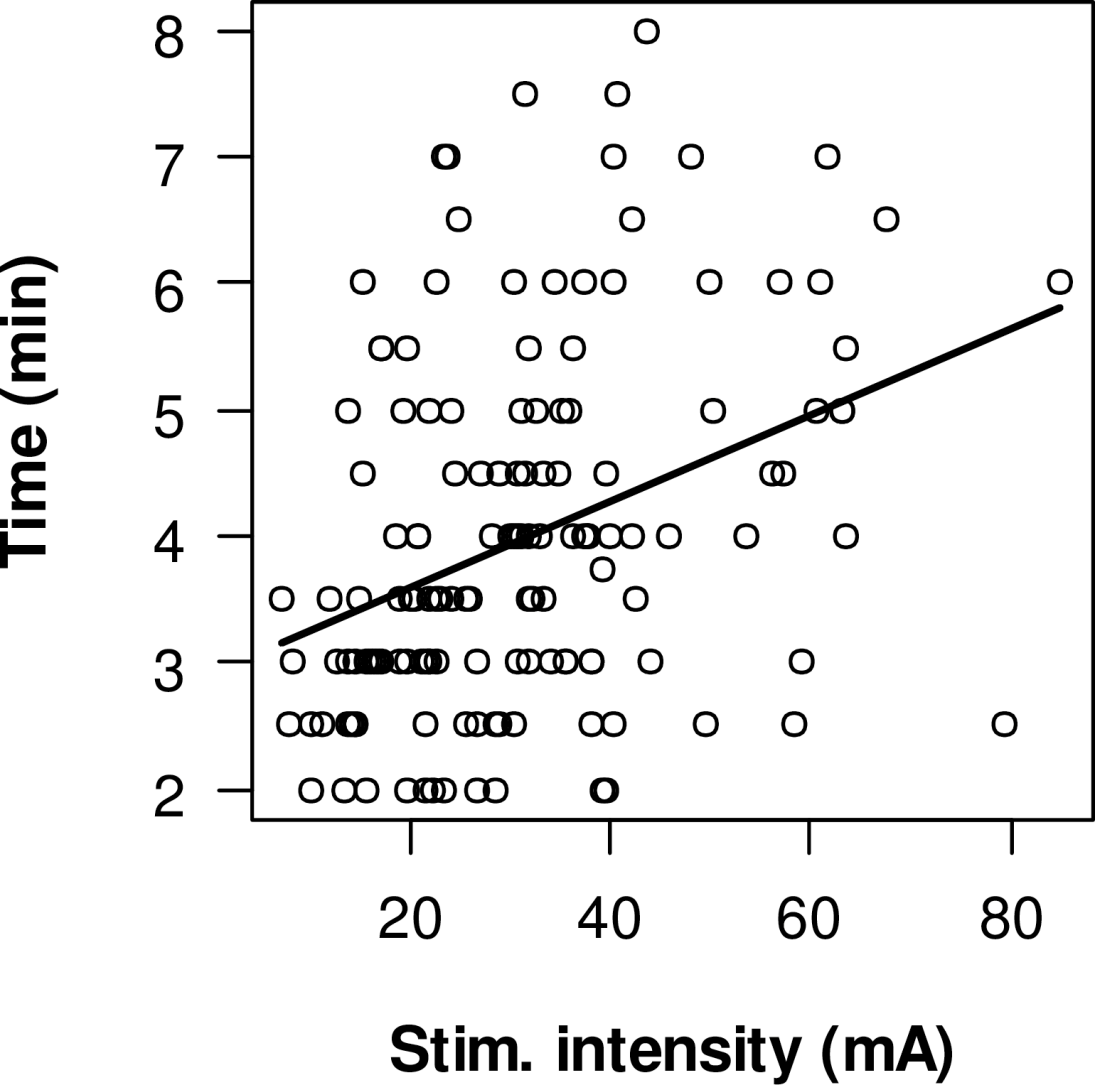
Correlation between duration of measurements and maximum stimulus intensity for all 6 muscles.

Longer duration of MUNIX measurements were correlated with higher stimulation intensities. (Fig 3) Linear regression of all measurements revealed a significant correlation (p<0.001) between higher stimulation intensities and longer duration of measurements. Each increase of 10 mA is estimated with 0.34 minutes longer duration. This was also true when excluding all measurements with very high stimulation intensities > 50 mA (p<0.001, 0,46 min per 10mA increase).

## Discussion

Reliability and accuracy of measurements is a key feature of any biological marker of disease. While this is relatively easily determined and common in laboratory medicine, it is much more challenging with physiological measures as these factors depend on both the subject’s and rater’s performance. Moreover, individual subjects cannot easily been sent to different laboratories. A “round robin test” is commonly used to evaluate reliability of measurements for biochemical and other “wet biomarker” laboratory tests between centres. [10, 13] This study is unique in that for the first time both reliability and accuracy of an electrophysiological measurement (MUNIX) was determined in a “round robin” setup.

The most important finding in this study it that the COV both within raters (intra-rater variability) and between raters (inter-rater variability) is equal or below 20%. The only exceptions are the AH which revealed the highest inter-rater COV for CMAP and MUNIX, and the intra-rater COV for EDB MUNIX. This reliability compares favourably with e.g. “wet biomarkers” of neuronal death and axonal damage like neurofilament (NF) proteins in cerebrospinal fluid, which exhibited an inter-lab COV of 59%. [14] Given that for biomarker qualification processes, as implemented by the FDA and European Medicines Agency (EMA), performance characteristics are also an important factor (www.fda.gov), we propose that regardless of the character of the biomarker (dry or wet), studies of inter and intra-rater reliability should be mandatory before such measures are taken up into clinical trials. [15, 16] Our study also provides evidence that reliability tests—as part of a quality control process–can be studied with reasonable costs and effort.

Previous data have suggested that intra- and inter-rater test-retest reliability of the MUNIX method is dependent on individual rater’s experience. [1, 2, 4–6, 17] In two multicentre MUNIX studies in healthy subjects and ALS patients, test-retest variability decreased in the second study in the same raters. [5, 6] In this study, no significant difference between trained raters less familiar with the MUNIX method and raters with several experiences in MUNIX was observed, suggesting that the method itself is robust and can be easily adopted. General electrophysiological practice seemed not to influence MUNIX reliability. However, all participants had several years of electrophysiological experience and at least one intense whole-day training course (theoretic aspects and hands-on training). This suggests that with appropriate training, MUNIX might be adopted with sufficient reliability in EMG labs.

It would be desirable to perform the same study setup with an ALS patient. However, for ethical reasons it seems inappropriate to perform 144 measurements in a patient over 2 days. From previous studies it is known that test-retest reliability is similar in ALS patients compared to healthy subjects. [5, 18] This suggests that this method can be applied reliably in ALS patients,

The AH muscle showed a tendency of lower CMAP and greater range of MUNIX and CMAP values and therefore lower accuracy when applying a hypothetical reference value. One reason might be that CMAP amplitude over AH is generated by multiple muscles after supramaximal tibial nerve stimulation and SIP recordings are mostly performed with voluntary toe flexion, as exclusive abduction of the hallucis is rarely obtainable. [19] It has also been demonstrated that MUNIX values are dependent of the direction of movement, which in total makes this muscle comparably unfavourable. [5, 20, 21]

The relative high MUNIX variability of the EDB muscle is most likely caused by comparably low absolute values. The volunteer exhibited a clearly damaged and atrophic EDB on the contralateral side; consequently, a bilateral (and before that date unrecognised) damage of the distal motor branch of the deep peroneal nerve might be the reason.

Single measurements were generally fast to perform in less than 5 minutes, with no significant difference between the experienced and less experienced group. We found a significant correlation of longer duration of measurements with increased stimulation intensities. This was visible in all 6 muscles, particularly in the biceps muscle, as electrical stimulation of the musulocutaneous nerve solely without co-stimulation of adjacent nerves is technically challenging. The most likely explanation is that raters, who had difficulties optimizing electrode position for maximum CMAP amplitude or finding the optimal stimulation electrode placement, tended to use higher electrical stimulation to ensure supramaximal nerve stimulation.

There are some limitations of this study. First, the test subject was not the typical volunteer and already familiar with this method. A “learning effect” seems possible, as MUNIX needs active cooperation of the test subject and so the study volunteer may have provided more consistent recruitment patterns than a typical study participant. Additionally, the environmental conditions were the same for all raters (EMG equipment and software, recording electrodes, filter settings), which might not be always the case in multicentre trials. Furthermore, less experienced raters performed measurements during or immediately after the training session. It is unclear, if the performance of these raters will persist when returning to their own EMG laboratory. In the aforementioned SOPHIA project, several raters failed to pass the qualification process at the first attempt. Therefore, we recommend continuous practice of this method prior to a reliability qualification process. The same would apply before this method is utilized in clinical trials, like in a previous MUNE study. [22]

## Conclusion

In conclusion, quality control of MUNIX shows that this is a reliable and robust electrophysiological method with high accuracy. Our data suggest that experienced neurophysiologists can easily utilize this method after appropriate training. Round robin tests can be implemented with reasonable effort to neurophysiological techniques.

## Supporting Information

S1 Table Raw data. Units of parameter: „time”= minutes; “CMAP” = mV; MUSIX = Motor Unit Size Index (μV); “stim” = stimulation intensity in mA; yellow fields = missing data.
(DOCX)
