# Supplementary material for: Quality Control of Motor Unit Number Index (MUNIX) Measurements in 6 Muscles in a Single-Subject “Round-Robin” Setup
Source: PLoS One. 2016 May 2;11(5):e0153948. doi: 10.1371/journal.pone.0153948 (PMC4852906; doi:10.1371/journal.pone.0153948)
Supplement: S1 Table — Units of parameter: „time”= minutes; “CMAP” = mV; MUSIX = Motor Unit Size Index (μV); “stim” = stimulation intensity in mA; yellow fields = missing data. (DOCX) [file pone.0153948.s001.docx]

Units of parameter: „time“ = minutes; “CMAP” = mV; MUSIX = Motor Unit Size Index (µV); “stim” = stimulation intensity in mA; yellow fields = missing data
